# Evaluating Large Language Models’ Ability Using a Psychiatric Screening Tool Based on Metaphor and Sarcasm Scenarios

**DOI:** 10.3390/jintelligence12070070

**Published:** 2024-07-21

**Authors:** Hiromu Yakura

**Affiliations:** Max–Planck Institute for Human Development, 14195 Berlin, Germany; yakura@mpib-berlin.mpg.de

**Keywords:** large language model, sarcasm, metaphor

## Abstract

Metaphors and sarcasm are precious fruits of our highly evolved social communication skills. However, children with the condition then known as Asperger syndrome are known to have difficulties in comprehending sarcasm, even if they possess adequate verbal IQs for understanding metaphors. Accordingly, researchers had employed a screening test that assesses metaphor and sarcasm comprehension to distinguish Asperger syndrome from other conditions with similar external behaviors (e.g., attention-deficit/hyperactivity disorder). This study employs a standardized test to evaluate recent large language models’ (LLMs) understanding of nuanced human communication. The results indicate improved metaphor comprehension with increased model parameters; however, no similar improvement was observed for sarcasm comprehension. Considering that a human’s ability to grasp sarcasm has been associated with the amygdala, a pivotal cerebral region for emotional learning, a distinctive strategy for training LLMs would be imperative to imbue them with the ability in a cognitively grounded manner.

## 1. Introduction

Since Aristotle’s time (Kenny 2013; Semino and Demjén 2016), figurative language—encompassing metaphors and irony—has been regarded as one of the most sophisticated aspects of human communication. Its realm of influence extends beyond the confines of literature, permeating everyday discourse, where its role is profound. For example, in casual phone conversations, figurative language occurs roughly once every 90 words (Bavelas et al. 2008). Hence, the ability to decipher figurative language is considered a pivotal objective in linguistic development (Willinger et al. 2017).

However, children possessing specific traits are recognized to grapple with challenges in comprehending such figurative language (Chahboun et al. 2021; Mention et al. 2024), as evidenced by studies suggesting distinct language processing patterns (Gold and Faust 2010; Vulchanova et al. 2019). Particularly, the comprehension of sarcasm in relation to Autism Spectrum Disorder (ASD) and other developmental disorders has received significant attention (Fanari et al. 2023; Happé 1995; Kalandadze et al. 2016). Some researchers have associated this with the point that previous studies comparing children with ASD to typically developing peers often did not consider matching groups based on general language skills, suggesting that observed difficulties in understanding sarcasm might stem from general linguistic abilities (Fuchs 2023; Gernsbacher and Pripas-Kapit 2012). However, it is also reported that individuals that possessed the linguistic IQ to comprehend metaphors still struggled with grasping sarcasm (Adachi et al. 2004, 2006). While its precise etiology remains elusive, individuals with the condition previously known as Asperger syndrome^1^ are known to manifest reduced connectivity in the amygdala, the cerebral region governing emotions (Baron-Cohen et al. 2000; Wang and Li 2023). Considering that such compromised connectivity of the amygdala impacts the cultivation of the *theory of mind* (Heyes and Frith 2014) in individuals with Asperger syndrome (Baron-Cohen et al. 1999), Adachi et al. (2006) postulated a potential commonality in the impediments to sarcasm comprehension. They subsequently proposed a screening test, the Metaphor and Sarcasm Scenario Test (MSST) (Adachi et al. 2004, 2006), to distinguish children with the condition then referred to as Asperger syndrome from those with akin symptoms, such as attention-deficit/hyperactivity disorder and high-functioning autism. This test measures comprehension of metaphors and sarcasm using five targeted questions for each category. The resultant scores were leveraged to delineate a distinct subgroup of children who were likely to be diagnosed with Asperger syndrome, portraying a substantial disparity between metaphoric and sarcasm comprehension.

Meanwhile, burgeoning advancements in large language models (LLMs), such as ChatGPT (OpenAI 2022), suggest an escalating potential for these models to undertake communications with humans (Wei et al. 2022). Under this trajectory, a precise understanding of these models’ limitations is deemed crucial not only for facilitating their communications with humans, but also for guiding their further development. Accordingly, this study utilizes the MSST to scrutinize the aptitude of recent LLMs in comprehending metaphors and sarcasm.

### 1.1. Previous Research

The elucidation of the emergent capabilities of LLMs (Wei et al. 2022) has enthralled the attention of numerous scholars. While much research has focused on assessing the extent to which LLMs can supplant human intellectual tasks (Atarere et al. 2024; Bubeck et al. 2023; Eloundou et al. 2023; Guo et al. 2023; Kim et al. 2024; Kung et al. 2023), such as medical decision making (Atarere et al. 2024; Kim et al. 2024; Kung et al. 2023), several studies explored the depth of LLMs’ grasp of human emotions and nuanced communications (Aghazadeh et al. 2022; Hagendorff 2023; Hagendorff et al. 2022; Kosinski 2023; Loconte et al. 2023; Marchetti et al. 2023; Nematzadeh et al. 2018; Safdari et al. 2023; Trott et al. 2023; Ullman 2023). Particularly, in studies probing their “theory of mind” through standardized tasks (Kosinski 2023; Marchetti et al. 2023; Nematzadeh et al. 2018; Trott et al. 2023; Ullman 2023), Kosinski (2023) reported that GPT-4 (OpenAI 2023) exhibits abilities akin to those of an 8-year-old human. However, research focusing on metaphors has been scant (Aghazadeh et al. 2022; DiStefano et al. 2024; Ichien et al. 2023; Loconte et al. 2023), with only a few reports highlighting their “superior” performance to humans (Ichien et al. 2023; Loconte et al. 2023) or their potential role as an automated assessor in creativity scoring (DiStefano et al. 2024), and does not incorporate a comparative aspect with sarcasm.

On the other hand, research efforts from an engineering perspective have addressed the enhancement of metaphorical and sarcasm comprehension in language models (Chakrabarty et al. 2022; Lal and Bastan 2022; Liu et al. 2022; Potamias et al. 2020; Prystawski et al. 2022; Sravanthi et al. 2024; Su et al. 2020; Yaghoobian et al. 2021). For instance, proposals have been posited to train dedicated models to detect metaphors (Su et al. 2020) and sarcasm (Potamias et al. 2020; Yaghoobian et al. 2021) within text and explain them (Lal and Bastan 2022). However, these approaches are crafted to leverage the semantical proximity between input texts and instances of metaphors and sarcasm in training datasets, thereby raising concerns about their applicability to novel metaphors and sarcasm. Alternatively, Prystawski et al. (2022) proposed a method to improve metaphor comprehension in LLMs by refining the instructions provided to the model (i.e., prompt). In addition, some datasets and benchmark tasks (Chakrabarty et al. 2022; Liu et al. 2022; Sravanthi et al. 2024) have been released to encourage the development of LLMs that are capable of comprehending figurative language. However, these efforts have not specifically focused on sarcasm comprehension, despite the knowledge from developmental psychology that revealed the contrastive distinction between metaphor and sarcasm understanding (Adachi et al. 2004, 2006). Therefore, this study evaluates LLMs specifically on sarcasm comprehension to enable discussions that stand on the intricacies of human language processing.

### 1.2. Research Questions

This study aims to deepen the understanding of LLMs’ capabilities in comprehending different forms of figurative language, specifically metaphors and sarcasm. To achieve this, the following research questions guide the investigation:

**RQ1**  How do current large language models (LLMs) compare in their ability to comprehend metaphors versus sarcasm?
**RQ2**  How does the ability to comprehend metaphors and sarcasm vary among LLMs with different parameter counts?

By addressing these questions, this study seeks to uncover specific challenges and limitations that LLMs face in processing figurative language. Furthermore, insights from developmental psychology will be integrated to highlight the cognitive distinctions between metaphor and sarcasm comprehension. Ultimately, this study aims to guide future advancements in model training and development to enhance their capabilities.

### 1.3. Significance of the Topic

Considering the eminence of figurative language in human communication, assessing LLMs’ comprehension abilities in this regard, particularly encompassing sarcasm, assumes paramount significance in augmenting their potential applications. Furthermore, the use of the standardized test for screening Asperger syndrome is substantive. Within this test, children without intellectual disability^2^ can attain high scores in both metaphorical and sarcasm comprehension (Adachi et al. 2004). On the other hand, the development of linguistic intelligence alone leads to the attainment of metaphorical comprehension, but not sarcasm (Adachi et al. 2006). This distinction would provide a new clue to understanding the evolution of LLMs. As Hagendorff (2023) alluded, we anticipate that a broader utilization of tools from developmental psychology elucidates the limitations and avenues for enhancement of LLMs.

## 2. Methods

### 2.1. Material

As mentioned above, the MSST was employed to assess LLMs’ comprehension of both metaphors and sarcasm. Gibbs (1994, 2011) stated that metaphor is a figure of speech where a word or phrase for a concept is used outside of its normal conventional meaning, often to express a similar concept. Here, metaphors can be categorized into conventional ones, which are commonly used and widely understood within a culture (e.g., “Love is a journey”), and novel ones, which involve unique or less common associations that challenge the individual to forge new semantic links (e.g., “We are driving in the fast lane on the freeway of love”). The MSST covers both types of metaphors to incorporate some challenging cases.

**Figure 1:**
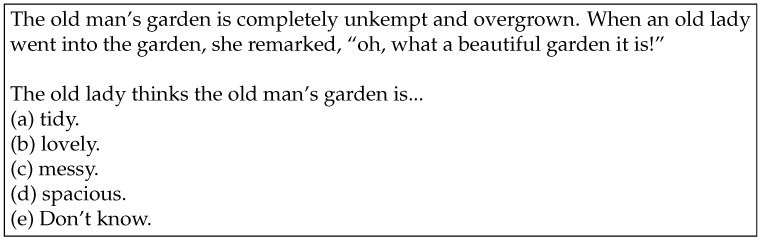
The actual content of Q8 (taken from Adachi et al. (2004)). No LLM selected the right choice, i.e., (c).

Irony, and more specifically sarcasm, is another complex form of figurative language. While irony involves expressing something contrary to what is meant, sarcasm often has a mocking or contemptuous tone (Garmendia 2018). According to (Sperber and Wilson 1981; Wilson and Sperber 2012), ironic speakers express their dissociative attitude towards an utterance or thought they are echoing, rather than simply presenting an attitude dissociative from the content. Meanwhile, Clark and Gerrig (1984) describe sarcasm as a form of pretense, where the speaker pretends to be an injudicious person, expecting the listener to recognize the insincerity. Given these points, understanding sarcasm requires inferring the speaker’s intention or judgment, which is attributable to the theory of mind, and thus can be challenging for children with the condition previously known as Asperger syndrome. From this point, the MSST employs scenarios that focus on this kind of sarcasm, as presented in Figure 1. We expected that using this test, grounded in figurative language studies, would enhance our understanding of LLM behavior relative to previous research on human communication.

### 2.2. Procedure

**Figure 2:**
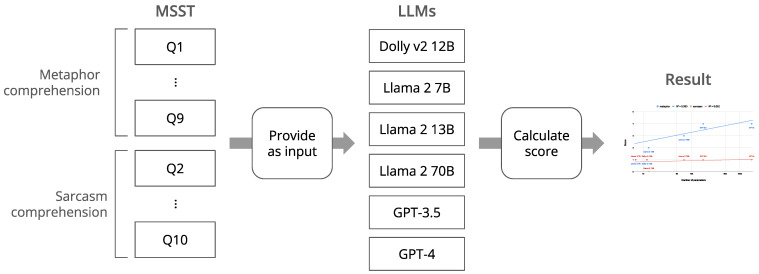
The procedure of this study.

The procedure of this study is illustrated in Figure 2. To juxtapose with previous research on the theory of mind (Kosinski 2023) and evaluate capacity variations based on differences in parameter count, we compared six LLMs, encompassing GPT-3.5 and GPT-4. The others were selected based on the availability of public access to the models and their performance with instruction-following tuning, namely Dolly 2.0 (Conover et al. 2023) and Llama 2 7B, 13B, and 30B (Touvron et al. 2023). For each model, we presented the MSST under the identical settings to conducting for human and calculated their correct answer rates. For each question, we provided the models with an instruction sentence and an explanation of the context and offered five choices, from which one had to be selected. Each correct response is counted as a point, with a maximum score of 5 for metaphor and sarcasm comprehension. Notably, no human participant was engaged in this study because Adachi et al. (2004) provided the scores of actual children, which enables us to compare their ability with those of the LLMs. Also, the source code necessary for replication, encompassing model version specifications, is publicly accessible at doi:10.5281/zenodo.10981763.

## 3. Results

**Figure 3:**
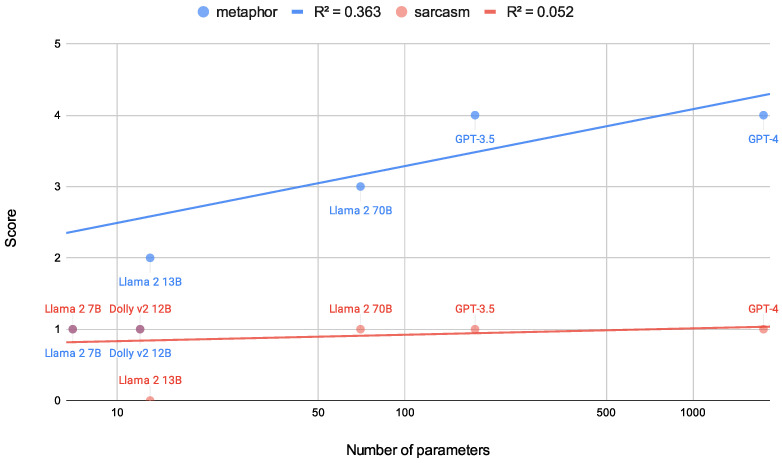
The comparison of the scores of LLMs on the MSST and the number of their parameters. Note that the parameter count of GPT-4 is not officially announced and is based on an online article (Schreiner 2023).

The results are presented in Table 1 and Figure 3. In terms of metaphor comprehension, it was observed that scores increased with an escalation in the number of parameters of the LLMs. Here, Adachi et al. (2004) reported a mean score of 4.1 for 199 children aged 8–10 years classified as without intellectual disability. In this context, we can conclude that GPT-3.5 and GPT-4 exhibited the ability to grasp metaphors that are equivalent to those of 8 to 10-year-old children. Note that this result corroborates Kosinski’s assessment of GPT-4’s inference capability based on the theory of mind (Kosinski 2023), which posited an aptitude akin to that of an 8-year-old. In addition, this is consistent with the report from Sravanthi et al. (2024), in which LLMs exhibited low performance for sarcasm understanding among various language understanding tasks, especially in comparison to human performance.

Conversely, with regard to sarcasm comprehension, none of the LLMs managed to answer more than a single question correctly, as shown in the even-numbered questions in Table 1. For instance, none of the LLMs could provide a correct response to Q8^3^ (see Figure 1). We may infer that it would not pose an incredibly daunting challenge for individuals possessing a fundamental conception of sarcasm, given that the average score for the 199 children was 3.3 (Adachi et al. 2004). However, both GPT-3.5 and GPT-4 opted for “(b) lovely,” yielding a score lower than the average of 1.8 achieved by 66 children diagnosed with Asperger syndrome (Adachi et al. 2006).

Here, since the MSST is published as a part of the existing paper (Adachi et al. 2004), we recognize the possibility that its questions might have been included in the LLMs’ training data, as other benchmark datasets have experienced (Golchin and Surdeanu 2024; Li and Flanigan 2024). However, if this were the case, it would be unusual for the models to comprehend only the metaphoric scenarios while scoring poorly on the sarcastic ones, suggesting that the questions might have been unfamiliar to the models. Furthermore, even assuming the questions were part of the training data, the results interestingly suggest that the models face difficulties in processing sarcasm, although they can effectively memorize metaphors. This underlines the apparent difficulty these models faced in comprehending sarcasm, irrespective of whether the MSST questions were included in their training data.

## 4. Discussion

Our investigation revealed that, whereas recent LLMs have substantially heightened their capacity for metaphorical comprehension with increased parameters, they still grapple with sarcasm comprehension. For humans, such a disparity is a distinctive trait associated primarily with individuals with the condition previously referred to as Asperger syndrome, and various explanations have been proposed, focusing on the distinct cognitive attributes characterizing this group (Loukusa and Moilanen 2009).

One explanation highlights that, while metaphor comprehension is driven by linguistic intelligence, sarcasm comprehension necessitates emotional intelligence, which involves recognizing and interpreting emotions and social cues (Adachi et al. 2004, 2006). Metaphors can often be understood by recognizing the discordance between their content and factual reality, which relies on cognitive processes that develop relatively early in life. For instance, children can understand basic metaphors by the age of three (Di Paola et al. 2020; Pouscoulous and Tomasello 2020). In contrast, as previously mentioned, sarcasm often involves content that remains plausible and requires the listener to detect the incongruity between the speaker’s literal words and their actual intent, which is inferred from the context (Clark and Gerrig 1984; Sperber and Wilson 1981; Wilson and Sperber 2012). This process involves the higher-order theory-of-mind ability to understand that others have beliefs, desires, and intentions different from one’s own (Happé 1993). Specifically, Mazzarella and Pouscoulous (2021, 2023) suggest that sarcasm comprehension depends on the emergence of vigilance towards deception, specifically “second-order epistemic vigilance,” which refers to the ability to assess others’ abilities to scrutinize deception. This explains why even children with typical development struggle with sarcasm comprehension until at least six years old (Filippova and Astington 2008; Pexman 2023). Consequently, this point presents a formidable challenge for individuals with the condition akin to Asperger syndrome, who often exhibit less performance in inferring others’ beliefs in the theory of mind tasks (Baron-Cohen et al. 1999, 2000; Wang and Li 2023).

Another explanation is rooted in the concept of weak central coherence (Frith 1989, 2008), which is prevalent in individuals with the condition previously referred to as Asperger syndrome (Jolliffe and Baron-Cohen 2000; Le Sourn-Bissaoui et al. 2011). They are said to exhibit a tendency to process information locally rather than globally, struggling to integrate information from multiple sources to derive context-dependent meanings (Frith 1989, 2008). In other words, their cognitive processes are significantly influenced by the literal meanings of certain words or expressions from isolated segments of communication, hindering their comprehension of sarcasm. These discussions provide inspiration for devising novel approaches to enhance the performance of LLMs, particularly their capacity to understand sarcasm.

For example, while the current training method of LLMs being reliant on extensive text datasets is effective in improving their linguistic intelligence, it may be inadequate when fostering emotional intelligence. In such a scenario, supplementing the current training regime with a distinct kind of training data could amplify the acquisition, akin to how individuals who were diagnosed with Asperger syndrome resolve social challenges a posteriori through social skill training (Rao et al. 2007). However, given the scarcity of available scenarios of social skill training compared to the datasets used for LLM training, strategic efforts are requisite to construct novel large-scale datasets or adopt new data-efficient fine-tuning techniques like Low-Rank Adaptation (LoRA) (Hu et al. 2022). In addition, given that multimodal pre-training using visual information is effective for knowledge transfer in LLMs (Muraoka et al. 2023), constructing multimodal datasets that include facial expressions may also prove effective, as it can enable capturing nuanced human emotion via pre-training as a vision large language model.

Furthermore, our findings can be associated with the fact that the majority of recent LLMs, including those examined in this study, are obtained using instruction-based tuning methods, such as InstructGPT (Ouyang et al. 2022). The popularity of these tuning methods stems from their effectiveness in enabling LLMs to follow user instructions accurately and avoid biased responses. However, it is conceivable that such tuning processes might impede the models’ ability to gauge human subjective judgments—a critical aspect in comprehending sarcasm, as highlighted earlier. This is because bias inherently intertwines with subjective judgments (Gilovich et al. 2002), and suppressing potentially biased outputs would inadvertently constrain inferences pertinent to human subjective judgment. From these points, it would be imperative to explore prudent strategies that enable LLMs to infer human subjective judgment while restraining biased outputs. Addressing this delicate balance can facilitate LLMs in engaging users through nuanced figurative language in witty dialogues.

Notably, it is known that LLMs are susceptible to misdirection by unrelated contextual information provided in a small portion of prompts (Shi et al. 2023). This raises the intriguing possibility that cognitive processes akin to those observed in individuals who were diagnosed with Asperger syndrome might influence their ability to comprehend sarcasm. Nevertheless, as a limitation of this study, it is imperative to consider that the behavior of LLMs may not directly mirror human neurological functions, even though high-level commonalities have been observed (Caucheteux et al. 2023; Evanson et al. 2023). We also need to mention that our results did not involve statistical tests due to the difficulty of forming a participant pool with LLMs and can be updated with future advancements in LLMs. Nonetheless, we believe that insights cultivated in developmental psychology can play a pivotal role in unraveling the behavior of LLMs, as exemplified by this study.

## 5. Conclusions

This study aimed to evaluate the capabilities of LLMs in comprehending figurative language, specifically metaphors and sarcasm. Specifically, we used the MSST and revealed that while LLMs show improved performance in metaphor comprehension with an increase in model parameters, their ability to comprehend sarcasm remains significantly limited. Our approach of using the established screening test allowed us to find the alignment of this disparity with insights from developmental psychology. Based on this, this study discussed the potential importance of emotional and social context in training methodologies, highlighting the importance of focusing on sarcasm comprehension in LLMs to better understand their limitations and guide future advancements. Future research should continue to develop strategies to enhance the emotional intelligence of LLMs, ultimately improving their application possibility within the full spectrum of human communication.

## Figures and Tables

**Table 1:** The performances of LLMs on the MSST, in which the metaphoric scenarios are odd-numbered and the sarcastic scenarios even.

Model	Metaphor Score	Sarcasm Score	Questions									
			Q1	Q2	Q3	Q4	Q5	Q6	Q7	Q8	Q9	Q10
Dolly v2 12B	1	1			✓							✓
Llama 2 7B	1	1			✓			✓				
Llama 2 13B	2	0	✓								✓	
Llama 2 70B	3	1	✓		✓			✓	✓			
GPT-3.5	4	1	✓		✓		✓	✓	✓			
GPT-4	4	1		✓	✓		✓		✓		✓	
Children w/o intellectual disability	4.1	3.3	—									
(Adachi et al. 2004)												

## Data Availability

All the data and source code necessary for replication is available at doi:10.5281/zenodo.10981763.

## Notes

1 While Asperger syndrome has been subsumed under the broader category of Social Pragmatic Communication Disorder in the DSM-5 (American Psychiatric Association 2013), this paper uses the term in its original context as it pertains to the MSST. This is to maintain coherence with the terminology used in MSST, given that our focus is on analyzing the behavior of LLMs rather than diagnosing human conditions.

2 While we recognize the potential outdatedness and inadvisability of this term, for reference, we note that Adachi et al. (2004) used the label “children without mental retardation” in their results.

3 The content of all questions is available in the source code published at doi:10.5281/zenodo.10981763.
